# Mechanisms of Contrast-Induced Nephropathy Reduction for Saline (NaCl) and Sodium Bicarbonate (NaHCO_3_)

**DOI:** 10.1155/2014/510385

**Published:** 2014-04-15

**Authors:** W. Patrick Burgess, Phillip J. Walker

**Affiliations:** MD Scientific, LLC, 1214 Wareham Court, Charlotte, NC 28207, USA

## Abstract

Nephropathy following contrast media (CM) exposure is reduced by administration before, during, and after the contrast procedure of either isotonic sodium chloride solution (Saline) or isotonic sodium bicarbonate solution (IsoBicarb). The reasons for this reduction are not well established for either sodium salt; probable mechanisms are discussed in this paper. For Saline, the mechanism for the decrease in CIN is likely related primarily to the increased tubular flow rates produced by volume expansion and therefore a decreased concentration of the filtered CM during transit through the kidney tubules. Furthermore, increased tubular flow rates produce a slight increase in tubular pH resulting from a fixed acid excretion in an increased tubular volume. The mechanism for the decreased CIN associated with sodium bicarbonate includes the same mechanisms listed for Saline in addition to a renal pH effect. Increased filtered bicarbonate anion raises both tubular pH and tubular bicarbonate anion levels toward blood physiologic levels, thus providing increased buffer for reactive oxygen species (ROS) formed in the tubules as a result of exposure to CM in renal tubular fluid.

## 1. Introduction

Contrast-induced nephropathy (CIN) was recognized after the use of CM began in medicine in the early 1950s [1]. This iatrogenic injury is a primary concern when CM is used arterially or intravenously for any reason and is a leading cause of mortality and morbidity in modern medicine [2]. While the exact mechanism of kidney injury associated with CM and the prevention of that injury have been the focus of many studies and reviews, a complete understanding of the pathophysiology of CIN, its consequences, and its prevention has not been established [3, 4]. The two major theories for CM kidney injury are as follows: (i) CM induced renal vasoconstriction that leads to medullary hypoxia and (ii) direct tubular injury produced by the concentrated CM within tubular fluid [3]. While the exact cellular mechanism(s) responsible for CIN are undefined, both of the postulated theories involve the generation of ROS and subsequent tissue injury that is likely to be mediated by ROS [4–6]. It is probable that a combination of numerous negative influences acting in concert leads to the generation of ROS as the major common pathway leading to CIN.

Although a greater understanding of the pathogenesis of CIN is needed, it must be emphasized that this paper is restricted to a discussion of the prophylactic effects of isotonic Saline and isotonic sodium bicarbonate in subjects receiving CM. Discussions of pathogenesis of CIN appear elsewhere in this symposium and in recent reviews [4, 7]. Isotonic sodium chloride (Saline) or isotonic sodium bicarbonate (IsoBicarb) is recommended for the reduction of CIN [3]. This paper will first discuss the renal handling of Saline and the mechanisms of its contribution to CIN reduction. A similar discussion of IsoBicarb will follow.

## 2. Contrast Media: Physiology Background

(P-1) The toxicity of CM for various human cells in cell cultures is dependent on CM concentration and time of exposure [8–11]. CM is most highly concentrated in the vessel into which the CM is injected and in the kidney, which excretes the vast majority of the CM [12, 13].

(P-2) With an injection of 100 mL of water soluble CM, such as iodixanol-320, in an 80 kg subject, one can estimate that the extracellular concentration of CM-bound iodine will promptly reach about 1.5 mg of iodine/mL by distribution of the CM in the extracellular space [12], a concentration roughly 1/200th that of the injected CM. With relatively normal kidney function, that CM will be excreted with a half-life of about two and a half hours. In the setting of advanced kidney insufficiency, the excretion half-life may be more than 10 hours [13, 14].

(P-3) CM, molecular weight 600–1650, is cleared at roughly the same rate as estimated for the subject's glomerular filtration rate (GFR) and is not metabolized within the tubule or reabsorbed from the lumen [10, 11]. Therefore, following glomerular filtration, the concentration of the filtered CM increases from its serum concentration to a potential maximum of 10 to >100 times that of the serum CM, depending on tubular flow in the various sections of the tubule as the kidney responds to hormones and intrarenal mechanisms that control sodium and water excretion [15, 16].

## 3. Saline: Physiology Background

(P-4) Normal human kidneys excrete salts, low molecular weight water soluble compounds, and excess water. The glomerular filtration rate is roughly 100 mL/min/1.73 m^2^. GFR is not significantly influenced by volume expansion or urine flow rate, although volume expansion could alter renal hemodynamics leading to increased tubular flow rates and small increases in filtration [17].

(P-5) Renal tissue typically exhibits an oxygen gradient ranging from a partial pressure of about 50 mm Hg in the renal cortex to about 20 mm Hg in the medulla and to as low as 10 mm Hg at the tip of the papilla [18]. Since renal vasoconstriction is a commonly described effect of CM administration, these levels of oxygen are likely to be even lower in the CM affected areas of the kidney.

(P-6) Healthy humans with normal kidney function respond to acute changes in hydration with changes in urine flow and composition within 30 minutes, as demonstrated in an article that measured urine and serum responses to an oral administration of sodium bicarbonate [19]. Intravenous changes in hydration would be expected to demonstrate an effect even more rapidly.

(P-7) Subjects with chronic kidney disease (CKD) are at increased risk for CIN [20]. Diseased kidneys do not excrete salt, water, and bicarbonate anion as efficiently as normal kidneys, and the duration of their exposure to CM after a given dose is prolonged compared to subjects that have normal kidney function. Diseased kidneys have reduced numbers of functional nephron units, potentially chronically ischemic areas, and reduced renal adaptations. These abnormalities place chronically injured kidneys at risk for CIN irrespective of any prophylactic clinical maneuver to reduce CIN injury. Nonetheless, subjects with advanced renal insufficiency are protected by hydration with Saline or IsoBicarb [3], although complete elimination of CIN has yet to be demonstrated.

### 3.1. Concentration Changes of Filtered CM during Transit through the Nephron Tubules in Response to Saline Infusion

(a) We will consider a hypothetical 80 kg subject who consumes 4 grams of sodium per day and net fluid intake of 1000 mL per day in excess of extrarenal losses and undergoes CM administration for a procedure. The subject's kidneys will filter CM at roughly the same rate as the GFR; thus the filtered luminal fluid has the same CM concentration as the glomerular capillary CM concentration. Within the nephron tubule the luminal CM concentration will increase from the filtered serum CM level to roughly 144 times that level as the tubules reabsorb salt and water (urine flow is 1000 mL/day or 0.69 mL/min; thus maximum concentration of filtered CM within the tubules is 100/0.69 or 144 times the serum CM concentration). A major portion of this concentration increase occurs in the proximal tubule. Because of passive water extraction within the descending limb of the Loop of Henle, the luminal CM concentration rises steadily as the CM approaches the tip of the Loop of Henle. Then depending on the relative water intake, that concentration will either remain the same or further increase depending on antidiuretic hormone (ADH) effects on distal and collecting tubules.

(b) If Saline is infused at 1 mL/kg/hr in this hypothetical subject for a period long enough to establish the excretion of the infused sodium and water, the concentration of the filtered CM through the nephron is lowered, reaching a maximum concentration of about 49 times the serum CM level. Nonetheless, the total CM amount filtered by each nephron remains the same regardless of the tubular flow rate (assuming that the Saline salt and water are excreted at the same rate as infused, the urine flow will be 1 mL/kg/hr × 80 kg/60 min/hr plus 0.69 mL/min or 2.03 mL/min. The maximum concentration within the tubules is then 100/2.03 or 49 times the serum CM concentration).

(c) If Saline is infused at 5 mL/kg/hr (i.e., 400 mL/hr) in this same subject, the concentration of the filtered CM is likely to reach a maximum of only 13 times the serum CM levels because of the marked increase in tubular flow rates throughout the kidney (assuming that the Saline's salt and water are excreted at the same rate as infused, the urine flow will be 5 mL/kg/hr × 80 kg/60 min/hr plus 0.69 mL/min or 7.36 mL/min. The maximum concentration is then 100/7.36 or 13 times the serum CM concentration).

### 3.2. Discussion of Saline

The literature evidence indicates that Saline is more effective than half normal Saline for the prevention of CIN [21]. Saline is also more effective than aggressive oral water intake alone [22]. High rates of Saline infusion (500–600 mL/hour) to closely match furosemide-stimulated urinary output have demonstrated a significant reduction in CIN [23]. These observations suggest that volume expansion with subsequent increased flow throughout the tubules should be a component of any hydration scheme intended to prevent CIN. If collecting tubule flow alone was sufficient to reduce CIN, water alone would suffice, a suggestion contrary to the described findings. Agents that temporarily increase the tubular flow but may induce subsequent dehydration (e.g., mannitol and furosemide without matching the urine output with saline replacement) have not been shown to reduce CIN [24]. Studies that used furosemide combined with matching urine output with Saline replacement have shown evidence of reduced CIN, suggesting that the increases in tubular flow must be maintained throughout the period of time of excretion of the CM. Furosemide has been described as raising medullary oxygen tension [25] which may reduce CIN, but only if the resulting diuresis is treated with Saline replacement.

Since Saline hydration alone has not been shown to significantly increase GFR, the tubular exposure time to any given CM dose as the CM is excreted by the kidney is the same for all degrees of hydration. The primary variable in terms of tubular CM exposure appears to be the intratubular CM concentration as affected by the hydration status of the subject and the subject's renal tubular responses to excrete the administered salt and water.

CIN is known to be related to the dose of CM during the exposure [2, 3, 7]. This observation is consistent with the notion that toxicity is related directly to CM concentration, which is determined by the dose of CM for a given subject and the subject's state of hydration.

Infusions of Saline that increase the tubular flow may also raise tubular pH slightly. The acid load that the kidney handles remains relatively constant over short periods of time. Therefore, raising the tubular volume will dilute the acid concentration and thus will lead to a slight rise in tubular pH.

## 4. Sodium Bicarbonate: Physiology Background

The physiology background listed for Saline is valid for IsoBicarb as well. The following background points are added to those described for Saline earlier.

(P-8) A normal human generates about 1 mEq of acid per kg per day as a result of protein catabolism. This acid load is excreted by the kidney, thus maintaining a stable systemic acid-base environment as the daily acid load is excreted in an acidic urine [26–28].

(P-9) As the proximal tubule reabsorbs sodium from the tubular fluid, bicarbonate anion is also reabsorbed in a reaction catalyzed by the presence of carbonic anhydrase in the proximal tubule brush border. With falling bicarbonate anion concentration, the pH within the proximal tubule drops to the range of 6–6.5 as bicarbonate anion levels fall to 6–8 mEq/L near the end of the proximal tubule [26–29].

(P-10) As fluid exits the proximal tubule and flows through the descending Loop of Henle, passive water removal concentrates the tubular fluid from a bicarbonate anion concentration of 6–8 mEq/L back to about 24 mEq/L and to near normal blood pH [26–29].

(P-11) Further absorption of bicarbonate anion occurs in the distal tubule leading to a near zero bicarbonate concentration and urine pH of <6 (dependent on the metabolic load of protein), buffered and supported by phosphates and ammonium to allow the kidney to excrete the typical acid load associated with protein metabolism [26–28]. However, the distal and collecting tubules are apparently less susceptible to CM injury than other portions of the nephron tubule despite the high CM concentration and low tubular pH. Perhaps structural differences such as the presence of “tight junctions” between the distal and collecting tubule cells or differences in the brush borders account for this apparent resistance to CIN [30].

### 4.1. Discussion of pH and Bicarbonate Anion Concentration Changes within Tubules

Based on the physiological background and in the absence of an alkali rich diet, the pH and bicarbonate anion levels in tubule fluid drop with transit through the proximal tubule and then the luminal pH increases back to nearly normal blood levels in the loop lumen at the tip of the papilla only to drop again with transit through the thick ascending limb of the loop and the distal tubule.

Pathologic evidence indicates that the initial injury associated with CIN occurs in the outer medullary section of the kidney in the medullary thick ascending limb (mTAL) of the Loop of Henle with less injury observed in another component of the outer medulla, the convoluted proximal tubule [4, 5]. CM concentrations within the tubule rise as salt and water are absorbed with flow through the proximal tubule and continue to rise flowing to the thick ascending limb. In the outer medulla segment of the kidney oxygen tension is low and pH is also low. Since the oxygen partial pressure is lowest at the tip of the papilla [18], oxygen content alone is unlikely to be the sole explanation of the observed medullary injury from CM. The cells of the thin Loop of Henle may be particularly resistant to oxidative injury, perhaps employing anaerobic metabolism to a greater extent than most other cells, although a protective effect of a near normal bicarbonate anion concentration cannot be excluded. The pH in the lumen of the mTAL typically drops back to the 6 range [26–28].

The increased sensitivity of the mTAL to CM injury may be a combination of (1) low pH, (2) high oxygen supply requirement secondary to active solute transport, and (3) reduced oxygen supply resulting from vasoconstriction that follows CM administration. Indeed, in vitro cell culture studies using several human and animal cell lines show that the apoptosis (programmed cell death) that occurs following free radical generation is markedly accelerated in an acid environment [31, 32]. Thus, when pH and bicarbonate anion levels are increased in kidney tissue there may be attenuation of free radical damage within the kidney, irrespective of the source of ROS.

Although the mechanism beyond its volume-expanding effects by which sodium bicarbonate might further reduce CI-AKI remains poorly defined, it has been postulated that sodium bicarbonate infusion may decrease generation of free radicals mediated by the Haber-Weiss reaction by increasing tubular pH [33]. The Haber-Weiss reaction is most active at lower pH levels. Sodium bicarbonate infusion may also scavenge the potent oxidant peroxynitrite, produced via a nitric oxide-mediated pathway [34]. Reactive oxygen species activate cytokine-induced inflammatory mediators, resulting in damage to proximal tubular cells, and it is likely that the activation of these mediators is influenced by tissue hypoxia and medullary acidosis.

While it may be very difficult to differentiate the volume-expanding effects of the two sodium salts from any direct effect on CIN in humans, animal studies with severe dehydration contribute to understanding of the role of bicarbonate. In a rat CIN model, rats were severely dehydrated using water restriction and diuretics and then exposed to CM or 1 mL of 8.4% sodium bicarbonate followed by CM [35]. In this study, 1 mL of 8.4% sodium bicarbonate was administered which represents 5 mEq of bicarbonate anion per kg but only 1 mL or 0.9% of the total animal estimated fluid volume. The pathology score at autopsy of the rat kidneys revealed reduced severe tubular damage scores in the bicarbonate treated animals: 71.4% versus 28.2%, CM alone versus 8.4% bicarbonate/CM, *P* = 0.02. Kidney tissue levels of glutathione, an oxidative stress marker, were also statistically reduced in the bicarbonate treated animals. These authors concluded that in this CIN rat model the bicarbonate protection was not solely related to sodium hydration, suggesting that free radical injury had been reduced as an effect of the bicarbonate administered.

Clinically, an alternative method of increasing bicarbonate anion levels in the proximal tubule and therefore throughout the renal tubules is with the administration of a carbonic anhydrase inhibitor, acetazolamide. One study compared acetazolamide to sodium bicarbonate therapy without controlling the IsoBicarb dose to match urine pH within the two cohorts [36]. This study in children with chronic kidney disease (CKD) showed a significant reduction in the incidence of CIN for acetazolamide treated subjects with a mean urine pH of 7.8 compared to the IsoBicarb treated subjects with a mean urine pH of 6.5. It is not known whether equivalent results would have been obtained by administering enough IsoBicarb to produce a urine pH of approximately 7.8. It should be noted that acetazolamide may produce extracellular volume reduction and produce systemic metabolic acidosis that could have other clinical effects. This study confirms that raising the proximal tubule pH and luminal bicarbonate anion levels (the primary effect of acetazolamide) has a beneficial effect on the CIN incidence in subjects with CKD, although the use of this diuretic may have other consequences.

Clinical experience suggests that IsoBicarb will add benefit, only if the dose is above a certain threshold. Since the first description of CIN reduction with IsoBicarb administration [33], a number of studies have confirmed the CIN benefit [37–41] while other studies have shown no benefit [42–46]. Studies that showed no benefit did not show any harm from the IsoBicarb. These neutral studies utilized a total dose of IsoBicarb that was lower (less than 1.5 mEq bicarbonate/kg) than the dose in those studies that showed statistically significant benefit (more than 1.5 mEq bicarbonate/kg). This raises the possibility that CIN benefit from IsoBicarb over Saline is likely to be dose related. One article with a higher dose of IsoBicarb did show a statistically significant reduction in CIN for the Saline cohort over the IsoBicarb cohort; however, the incidence of CIN for the Saline treated control cohort in this report was extremely low based on the control cohort's described risk factors [20, 47]. With this one exception, IsoBicarb when used in higher doses has demonstrated superior CIN results when compared to Saline.

## 5. Conclusions

As the above points indicate, several important factors are known to influence the development of CIN in human subjects:

- CM concentration within the renal tubules is determined by the dose of CM and by the rate of flow within the tubule itself, the latter dependent largely on the rate of sodium containing fluid administration;
- time or duration of exposure is a variable dependent chiefly on GFR and not easily altered by clinical maneuvers;
- pO_2_ varies in different portions of the kidney, with pO_2_ known to fall dramatically from outer cortex to renal papilla; the presence of regional variations in tissue oxygen content and the high oxygen demand placed on the mTAL segments to support solute transport functions at a time when oxygen availability is reduced by CM-induced vasoconstriction lead to hypoxia of the mTAL and favor production of increased levels of ROS;
- pH within the tubular lumen is determined primarily by bicarbonate anion concentration, a level that oscillates markedly from glomerulus to end of the collecting duct; the concentration of bicarbonate anion and therefore the pH of tubular fluid can be greatly affected by the administration of sodium bicarbonate;
- the primary cellular injury in CIN appears to be mediated by ROS generation;
- lower bicarbonate concentrations (low pH) accelerate ROS induced toxicity in cell studies.

Of the listed points, only (i) CM luminal concentrations and (iv) luminal pH are easily manipulated by therapeutic interventions. The above points and related clinical studies lead to these conclusions.

- Saline before, during, and after exposure to CM will produce an infusion rate-dependent increase in tubular fluid volume, reduction in CM intratubular concentrations, and slight increases in tubular pH; these lower tubular concentrations of CM should lead to reduced ROS formation and are the most likely mechanism of CIN reduction to be related to Saline infusion; therefore, the effect on CIN is likely to be Saline infusion rate-dependent as well and requires that the infusion be maintained throughout the period of CM excretion by the kidney.
- IsoBicarb infusion before, during, and after exposure to CM will produce the same effects of systemic volume expansion and increased tubular volume that follow Saline administration as described above, with the additional benefit of a substantial increase in the bicarbonate anion buffer throughout the renal tubule; low pH is known to accelerate cellular apoptosis in the setting of free radical formation, a toxic effect that may be ameliorated by raising the pH with bicarbonate.

Therefore, reduced CIN associated with IsoBicarb infusion appears to be at least partially if not largely related to an increase in filtered bicarbonate anion and subsequent increase in tubular bicarbonate anion concentration. The effect of IsoBicarb on CIN is likely to be infusion rate- or dose-dependent as is the case with Saline. IsoBicarb infusion is likely to show additional benefit when compared to Saline, only when adequate doses of IsoBicarb are administered prior to the CM exposure, that is, enough IsoBicarb to raise proximal tubular bicarbonate anion and pH to levels close to those found in blood, with the IsoBicarb infusion maintained after the CM exposure to keep renal bicarbonate anion levels raised while the CM is excreted.
